# Deep Learning‐Based Analysis of Gene Expression Data and Gene‐Related Information in Pediatric Surgical Oncology: A Scoping Review

**DOI:** 10.1002/cam4.71976

**Published:** 2026-05-22

**Authors:** Simon Berhe, Steffen E. Fuchs, Altuna Akalin, Alida F. W. van der Steeg, Steven W. Warmann, Myrthe A. D. Buser, Moritz Markel

**Affiliations:** ^1^ Department of Pediatric Surgery Charité‐Universitätsmedizin Berlin Germany; ^2^ Max Delbrück Center for Molecular Medicine in the Helmholtz Association (MDC) Berlin Germany; ^3^ Berlin Institute of Health at Charité—Universitätsmedizin Berlin Berlin Germany; ^4^ Department of Pediatric Oncology and Hematology Charité—Universitätsmedizin Berlin Berlin Germany; ^5^ German Cancer Consortium (DKTK), partner Site Berlin, a Partnership Between DKFZ and Charité‐Universitätsmedizin Berlin Berlin Germany; ^6^ Bioinformatics and Omics Data Science Platform Berlin Germany; ^7^ Princess Máxima Center for Pediatric Oncology Utrecht the Netherlands

**Keywords:** deep learning, gene expression, gene‐related information, pediatric surgical oncology

## Abstract

Deep learning (DL) methods may enhance analysis of complex gene expression data to aid in diagnosis and treatment planning for pediatric extracranial tumors. However, the literature regarding the application of DL to gene expression data in this field remains limited. This scoping review was based on the question “What is the current status of research in gene expression, gene‐related information and deep learning‐based analyses for pediatric surgical oncology*”*. We conducted a scoping review in accordance with the PRISMA‐ScR guidelines. A systematic search of PubMed, Scopus, and Embase was performed to identify studies applying DL models to gene‐related data in pediatric extracranial solid tumors. After deduplication, title and abstract screening and full‐text screening, nine studies met the inclusion criteria. Neuroblastoma was the most commonly studied tumor type (*n* = 6), with classification and survival prediction as applications. In general, the studies reported strong performance; however, external validation was rarely reported. Although the application of DL to gene‐related data in pediatric solid tumors remains in its infancy, current studies highlight the diversity and potential of approaches that could improve classification, prognostication, and the treatment of patients. The large variety of technical approaches reflects the ongoing process of adaptation to gene‐related data. Advancing this field will require larger datasets, consistent methodology, external, and prospective validation within a cross‐disciplinary setting.

**Abbreviations**
AI: artificial intelligence
AUC: area under the curve
AUPR: area under the precision–recall curve
DL: deep learning
DNN: deep neural network
INSS: International Neuroblastoma Staging System
LDA: lncRNA–disease association
lncRNAs: long non‐coding RNAs
ML: machine learning
NB‐MuSE: multi‐signature ensemble classifier
PPB: pleuropulmonary blastoma
RNA: ribonucleic acid
RNA‐seq: RNA sequencing
scRNA‐seq: single‐cell RNA sequencing
SRBCTs: small round blue cell tumors

## 1 Introduction

Cancer is still among the leading disease‐related causes of death in children [1]. Although diagnostic tools and prognostic models have improved, predicting outcomes in pediatric solid tumors remains challenging, especially in high risk scenarios [2]. For high‐risk or relapse cases, survival rates are still unacceptably low, necessitating new strategies [3]. In neuroblastoma, for instance, current staging relies heavily on imaging and clinical parameters, while the inclusion of new genetic markers may offer more precise outcome predictions [2].

Implementation of gene‐expression, gene‐related data and biomarker discovery have the potential to facilitate early diagnosis, precise tumor subtyping, and targeted therapy, especially for rare entities like pediatric solid tumors. Biomarkers such as *MYCN* and *WT1* are relevant for various tumor types including neuroblastoma, nephroblastoma, hepatoblastoma, and rhabdomyosarcoma, and the advent of high‐throughput platforms has generated extensive datasets [3, 4, 5, 6, 7]. With microarray, total and single‐cell RNA sequencing (scRNA‐seq), a wide variety of state‐of‐the‐art technical approaches exist, each with its own strengths and shortcomings. However, correlation to clinical parameters and patient outcome is often challenging, due to the huge extent and complexity of datasets that might develop [8].

**FIGURE 1:**
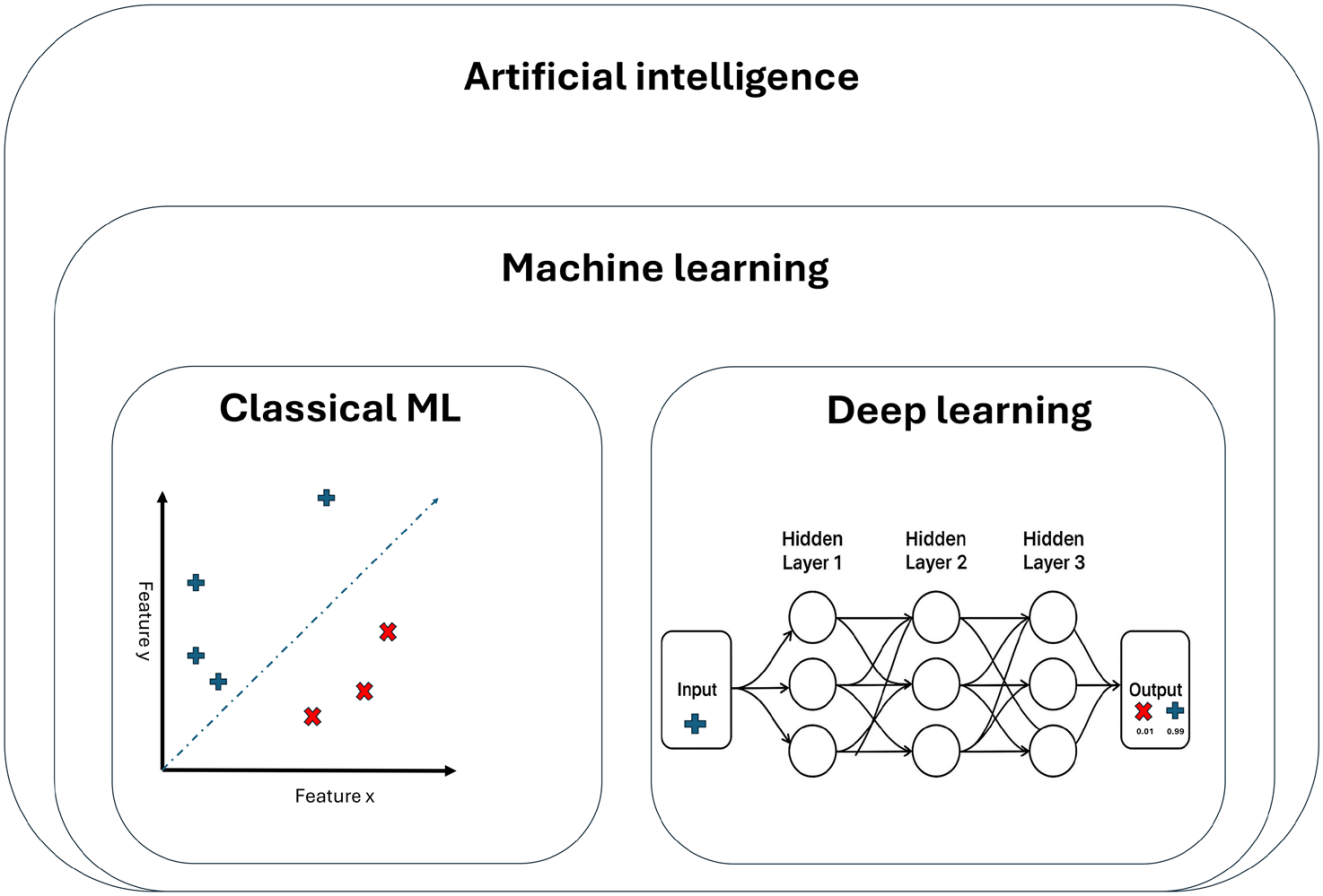
The relationship between artificial intelligence (AI), machine learning (ML), and deep learning (DL). ML is a subset of AI, aimed at the automation of tasks. This can be divided in classical machine learning, in which a relationship between features is extracted to perform a certain task, or deep learning, in which large computational networks are used to learn patterns without the need of identifying specific features. In this example, both the classical ML and the DL method are used to classify between × and +. The classical ML method used the relation between feature *x* and *y* to identify any of the examples. The DL method, for which the training is not depicted in the image, uses a neural network of three or more layers to determine the likelihood of the example being either × or +.

Artificial intelligence (AI) approaches, particularly machine learning (ML) and deep learning (DL), offer tools to decipher such complex data [9, 10]. ML is an umbrella term for computer algorithms that are able to learn, adapt, and draw inferences from data without specific instructions [11]. Classical ML techniques include methods like linear and logistic regression, clustering algorithms and support‐vector machines. DL is an advanced field within ML that utilizes neural networks, extensive algorithms of interconnected layers that resemble the human brain. DL, by definition, is a neural network with three or more hidden layers. Hidden layers extract patterns from data, ranging from simple tasks to increasingly complex operations as more layers are added [12]. This complexity enables DL to perform tasks and discover patterns in a wide range of applications, including complex and large‐scale datasets (Figure 1).

DL has shown promise in radiologic analysis for both adult and pediatric cancers [13, 14, 15]. Yeow et al. [16], for example, developed a DL model that predicted *MYCN* amplification from CT scans, enhancing outcome prediction for neuroblastoma patients. Although the results in radiology are promising, DL applications for gene‐related data in pediatric extracranial solid tumors remain sparse. DL can be trained on these data, which could ultimately lead to advances in the field, including more precise risk classification of tumors and prevention of misclassification and under‐ or overtreatment [17].

However, while numerous adult oncology studies utilize DL in gene‐related analysis, the pediatric‐specific literature is limited [9, 10, 18]. This review aims to fill this gap by mapping existing studies and highlighting potential starting points for application of DL and gene‐related information in pediatric extracranial solid tumors.

## 2 Materials and Methods

This scoping review was guided by the central research question: What is the current status of research in gene expression, gene‐related information and deep learning analyses for pediatric surgical oncology? To ensure methodological rigor, predefined inclusion and exclusion criteria were applied. The review process adhered to the PRISMA guidelines for scoping reviews (PRISMA‐ScR) [19].

### 2.1 Search Strategy

A broad, comprehensive search was performed in PubMed, Scopus, and Embase (October 2024), with search terms tailored for each database. Full queries are listed in Table S1. Duplicates were removed, and the remaining studies were screened (Tables S1–S3).

### 2.2 Inclusion and Exclusion Criteria

We included studies that investigated the application of DL to gene expression data in pediatric extracranial solid tumors. We did not include central nervous system tumors, as these are predominantly treated and investigated by pediatric neurosurgeons. In the context of this article, DL was defined as a neural network architecture comprising three or more hidden layers (i.e., layers other than the input and output layers). Accordingly, studies utilizing neural networks with only a single layer or fewer than two hidden layers (e.g., shallow neural networks) were excluded [12]. Studies employing related gene‐related information, such as epigenetic modifications (e.g., DNA methylation), were also considered for inclusion when applicable. A detailed overview of the inclusion and exclusion criteria is provided in Table 1.

**TABLE 1:** List of inclusion and exclusion criteria.

Criteria type	Description
Inclusion	Studies about extracranial, pediatric solid tumors
Inclusion	Studies about DL
Inclusion	Studies involving gene expression data, genes, methylation, and other gene‐related information
Exclusion	Studies about intracranial, pediatric solid tumors, or other non‐surgical oncologic diseases
Exclusion	Median age not reported in a non‐childhood specific tumor
Exclusion	Median age > 18 in a non‐childhood specific tumor
Exclusion	Classical ML or studies without ML/DL
Exclusion	No peer‐review or no original study
Exclusion	Other language than English
Exclusion	Study about radiology/imaging and DL (in case of combination with gene expression data full text screening decides)
Exclusion	Study about histology/pathology imaging and DL (in case of combination with gene expression data full text screening decides)
Exclusion	Study about clinical data (input) and DL (in case of combination with gene expression data full text screening decides)

### 2.3 Screening

Initial screening was conducted using ASReview (version 1.2.1), which employs an active learning approach to dynamically prioritize studies based on relevance [20]. As screening progresses, the algorithm continually updates the ranking of studies to enhance efficiency. The process was initiated by manually selecting three relevant and three irrelevant studies based on domain knowledge. To guide termination of the screening, we applied a combination of two commonly used stopping rules from the literature: first, a minimum of 33% of the total study pool had to be screened, and second, screening continued until 50 consecutive studies were labeled irrelevant [20]. In practice, both reviewers independently encountered over 1000 consecutive irrelevant studies after screening approximately 2000 abstracts each, and screening was terminated early. This constituted a deviation from the pre‐specified stopping rule of screening at least 33% consecutive irrelevant studies. Two pediatric surgeons (S.B. and M.M.) independently screened all titles and abstracts. Since the ASReview algorithm dynamically changes the order of studies, each reviewer may have been presented with different subsets. We defined non‐consensus as: first, one reviewer included a study while the other excluded it, or secondly, a study was included by one reviewer but not reviewed by the other. These cases were resolved through discussion. If one reviewer excluded a study that the other had not reviewed, this was interpreted as implicit consensus for exclusion. In cases of discrepancies, a technical physician (M.A.D.B.) was consulted. Subsequently, full‐text articles of the included studies were reviewed for final eligibility. Discrepancies were again resolved through discussion, with input from the technical co‐author as needed. Additionally, reference lists of the included studies were screened to identify potentially relevant articles not captured in the initial search.

### 2.4 Data Extraction

From each included pediatric study, we extracted key contextual information, including year of publication, tumor type, and primary analytic task (Table 2). Next, we reported technical information about data source, model architecture, and the number of input features, and performance metrics (Table 3). In cases where multiple DL models were evaluated within a single study, we reported the performance metrics of the best‐performing model. If both DL and classical ML models were compared, only the performance of the DL approach was included in our report. Lastly, a PROBAST risk‐of‐bias assessment was performed.

**TABLE 2:** Overview of the included articles with the relevant extracted information.

First author	Year	Tumor type	Task
Bandi	2023	Pleuropulmonary blastoma	Classification (tumor type)
Cornero	2012	Neuroblastoma	Classification, prediction (OS)
Feng	2021	Neuroblastoma	Prediction (OS; EFS)
Hosseiniyan Khatibi	2023	Wilms/rhabdoid tumor kidney	Classification (tumor type)
Li	2023	Osteosarcoma	Classification (tumor type), prediction (OS)
Pal	2007	Small round blue cell tumors: Neuroblastoma, non‐Hodgkin lymphoma, rhabdomyosarcoma, ewing sarcoma	Classification (tumor type)
Park	2019	Neuroblastoma	Classification (tumor type/stages)
Su	2023	Lung cancer, Neuroblastoma	Predicition of potential lncRNAs based biomarkers
Tranchevent	2019	Neuroblastoma	Prediction (death from disease, disease progression)

Abbreviations: AUC‐ROC, area under the receiver operating characteristic curve; EFS, event free survival; OS, overall survival.

**TABLE 3:** Overview of the included articles with the technical details.

First author	Data and source	Data type	Code and model data availability	Model	Performance (test set)	Number of features
Bandi	Public imaging data + DICER1‐AS1 gene (HGNC : HGNC : 43017)	CT, DX, CR, DICER1‐AS1 gene (HGNC : HGNC : 43017)	Code: n/a; data: on request	CNN (CT images + DICER‐1)	96% accuracy for DICER1‐DNA	N/A
Cornero	182 patients (4 cohorts; Netherlands; Germany; Italy; Japan GSE), 33 published prognostic gene signatures	Microarray gene expression, published gene signatures	Code: n/a; data: on request	Ensemble classifier (NB‐MuSE) incl. 20 gene signature‐based models, decision table	94% accuracy (dead/alive)	5–117 genes
Feng	721 samples, SEQC GSE49710 and E‐MTAB‐8248 and clinical data	Microarray	Code: n/a; data: on request	Supervised Deep Learning with Attention Mechanism (Vaswani et al.)	0.878–0.904 AUC‐ROC	172 gene features. Identification of lncRNAs
Hosseiniyan Khatibi	The cancer genome atlas (TCGA, messenger RNA profiles W 136, RT 63, micro RNA profiles: W 138, RT 78)	mRNA profiles, micro RNA profiles	Code: n/a; on request	Louvain, Boppana algorithm, self‐organizing deep auto encoder	93% accuracy miRNA, 97% accuracy mRNA	85 miRNAs, 31 mRNAs
Li	GSE14359, GSE99671, GSE126209 (training 39 sample), and GSE19276 (validation 23 samples)	Microarray	Code: n/a; on request	Random Forest (RF) and Artificial Neural Network (ANN)	Classification 0.987 AUC, prediction 0.629–0.691 AUC	9 gene targets
Pal	88 samples, 63 training (E 23, NB 12, NHL 8, RMS 20) 20 testing (E 6, NB 6, NHL 3, RMS 5), cDNA microarray	cDNA microarray	Code: n/a; data: n/a	Neural Network (multilayered perceptron (MLP)) and fuzzy clustering	(only training set, 100% accuracy)	7 gene targets
Park	GSE85047, 280 samples	Microarray	Code: n/a; data: n/a	Convolutional neural network (CNN)/MLP or other DNN architecture	AUC average 0.71, stages 0.58–0.8 AUC	13,901 genes
Su	lncRNA‐disease associations (LDA) model, 2 datasets (605 LDAs between 157 diseases and 82 lncRNAs. + 1529 LDAs between 190 diseases and 89 lncRNAs)	LDAs, lncRNAs	Code: n/a; data: on request	GCN/GAT/CNN, Ensemble of a deep neural network (DNN) + LightGBM (gradient boosting)	AUC 0.87–0.97	82 + 89 lncRNAs
Tranchevent	Zhang et al. 2014 (498 samples) for training and testing, Wang et al. 2006, 92 samples, for testing, Molenar et al. 2016, 88 samples for testing	Microarray, RNA‐seq	Code: available; data: available	Wilcoxon analysis/Patient Similarity Networks (PSN) are used to train DNN, comparison of performance with SVM and RF	DNN: 85%–87% accuracy, SVM/RF: 75%–82% accuracy	638 and 2196 features, 12–101 moduls of topographical feature sets

Abbreviations: CNN, convolutional neural network; DNN, deep neural network.

## 3 Results

**FIGURE 2:**
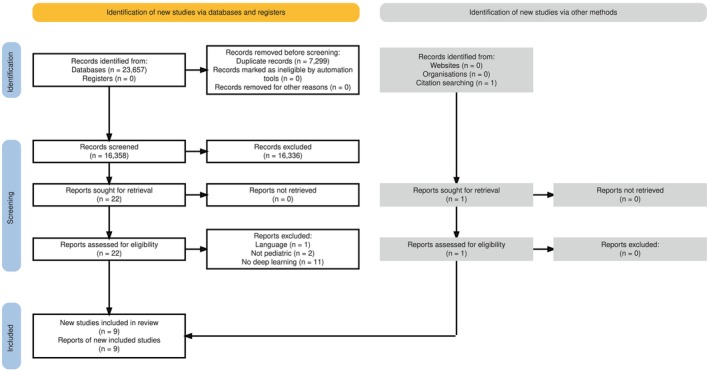
PRISMA flow diagram of the article screening. The workflow included an extensive search of the literature (identification), followed by several steps of screening. The search revealed 16,358 abstracts, and a final number of nine articles was included. AS Review reduced the manually screened number to ~2000 abstracts.

Using our predefined search strategy (Table S1), we identified a total of 23,657 records across three databases: PubMed (*n* = 1860), Scopus (*n* = 14,262), and Embase (*n* = 7535). After removing duplicates, 16,358 unique studies remained for screening (Figure 2; Tables S2 and S3). After each reviewer screened approximately 2000 abstracts, using ASReview, we encountered over 1000 consecutive irrelevant studies. Therefore, we adjusted our approach and terminated screening as no additional relevant studies were expected, likely as a result of our broad search strategy in combination with a narrow set of inclusion criteria [20].

**FIGURE 3:**
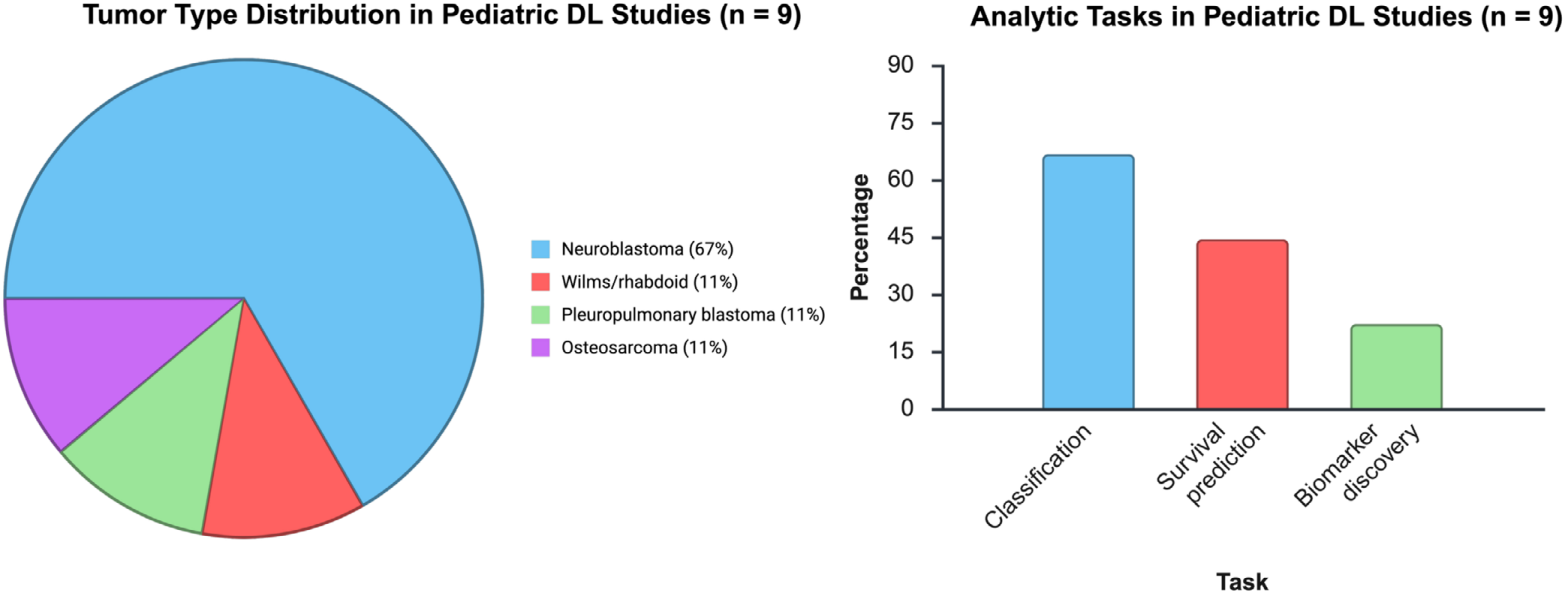
Distribution and Analytic Tasks of Pediatric DL–Gene Expression Studies: Panel A: Distribution of tumor types in pediatric DL studies (*n* = 9): Neuroblastoma (≈66.7%), Wilms/rhabdoid tumor (≈11.1%), pleuropulmonary blastoma (≈11.1%), and osteosarcoma (11.1%). Panel B: Breakdown of analytic tasks in pediatric DL studies: Classification (≈66.7% or *n* = 6), survival prediction (≈44.4% or *n* = 4), and biomarker discovery (≈22.2% or *n* = 2). Note that the categories are not mutually exclusive; studies might address multiple analytic tasks.

Title and abstract screening yielded 22 studies for full‐text review. Most full‐text exclusions were due to the use of classical machine learning models rather than DL architectures. Ultimately, nine studies met all inclusion criteria and were incorporated into the final analysis (Figure 2). One study was identified by screening the references of the included full‐text articles. An alphabetical overview of all included studies is presented in table 2 with technical details in table 3 [21, 22, 23, 24, 25, 26, 27, 28, 29]. Most studies (*n* = 6) utilized gene‐related data derived from neuroblastoma patients or neuroblastoma tumor samples. Two studies also included additional tumor types: non‐Hodgkin lymphoma, rhabdomyosarcoma, Ewing sarcoma (*n* = 1), and lung cancer (*n* = 1) [25, 27]. We further identified one study focused on Wilms tumor and rhabdoid tumor of the kidney, one study analyzing pleuropulmonary blastoma (PPB), and one that addressed osteosarcoma across both pediatric and adult cohorts. The oldest included study was published in 2007, while the majority were published in 2023 (Table 2, Figure 3). The PROBAST risk‐of‐bias assessment for all included studies can be seen in Table S4.

Six of the nine included studies investigated neuroblastoma. Cornero et al. [22] developed a multi‐signature ensemble classifier (NB‐MuSE) by integrating 33 previously published gene expression signatures, including three multilayer perceptron (MLP) neural networks. Their ensemble model, using a decision table algorithm, achieved an external validation accuracy of 94%, while the MLP models individually reached 80% accuracy [22]. Feng et al. [23] implemented a DL model augmented with an attention mechanism to predict neuroblastoma patient survival. Trained on two publicly available datasets (GSE49710 and E‐MTAB‐8248; total *n* = 721), the model achieved an AUC of 0.891 on the test set, outperforming two models using classical machine learning approaches. Additionally, the study identified 17 potentially relevant long non‐coding RNAs (lncRNAs) that participate in the regulation of important genes within the genetic network (hub genes) [23]. Pal et al. [25] used a combination of methods to identify a minimal biomarker panel from cDNA microarray data. Their study, based on 88 samples (63 training, 20 testing; 5 were later found to be outside of the inclusion criteria), achieved 100% accuracy in classifying among four subtypes of small round blue cell tumors (SRBCTs): neuroblastoma, non‐Hodgkin lymphoma, rhabdomyosarcoma, and Ewing sarcoma. The final model used just seven genes (FVT1, CDH2, FGFR4, AF1Q, NAB2, EHD1, and LSP1) [25]. Park et al. [26] applied a DL network to predict neuroblastoma stage according to the *International Neuroblastoma Staging System* (INSS). The model demonstrated moderate‐to‐high discriminative performance, with one‐vs‐rest AUCs for INSS stages ranging from 0.58 to 0.85, despite a relatively small dataset [26]. Su et al. [27] developed a model called LDAenDL, based on two lncRNA‐disease association (LDA) datasets (dataset 1: 605 LDAs between 157 diseases and 82 lncRNAs; dataset 2: 1529 LDAs between 190 diseases and 89 lncRNAs). LDAs already show documented connections of lncRNAs to specific diseases, which could offer new insights to complex diseases. The authors created a hybrid model combining a deep neural network (DNN) and LightGBM, to identify candidate lncRNA biomarkers for lung cancer and neuroblastoma. Although the study described high precision, recall, accuracy, F1‐score, area under the curve (AUC), and area under the precision and recall curve (AUPR) for the described model in classifying the LDAs, there were no specific metrics reported for the extraction of the top 20 associated lncRNAs in neuroblastoma [27]. Tranchevent et al. [28] utilized four datasets to show that a DNN reached high performance rates in outcome prediction of neuroblastoma patient samples of 85%–87% after training with previously created models of topographical feature sets of the data. This approach led to a reduction of features and higher performance compared to external SVM or RF [28].

Bandi et al. [21] used a deep convolutional neural network (DCNN) architecture for classifying pleuropulmonary blastoma (PPB) using CT imaging data and DICER1‐related RNA‐seq gene expression data. Of the 245,000 available imaging records, only 500 CT images were used (450 training, 50 validation). While the RNA‐seq‐based approach alone showed an accuracy of 95%, the integrated approach achieved 96%. The authors did not report the size of the DNA‐DICER1 dataset [21].

Hosseiniyan Khatibi et al. [29] applied a combination of filtering, graph‐based methods, and association rule mining to identify key mRNAs and microRNAs associated with Wilms tumor and rhabdoid tumor of the kidney to discriminate these kidney‐specific entities. Classification performance was high for both molecular types: miRNA‐based models achieved AUCs of 0.96 and 0.93, and mRNA‐based models reached AUCs of 0.95 and 0.98, with corresponding accuracies of 94%–97% [29].

Li et al. [24] constructed a diagnostic model for osteosarcoma using a combination of random forest and artificial neural networks to classify osteosarcoma based on gene expression. Based on multiple *Gene Expression Omnibus* (GEO) datasets (GSE14359, GSE99671, GSE126209, and GSE19276), they identified a nine‐gene panel (e.g., TNFRSF21, HSPB8, and TGFBR3) that differentiated osteosarcoma from normal tissue. The model achieved an AUC of 1.0 in the training set and 0.987 in external validation [24].

## 4 Discussion

Amid the growing adoption of DL in healthcare, its integration into pediatric oncology has emerged as a particularly timely and increasingly important area of exploration. This scoping review set out to systematically identify and evaluate studies that applied DL techniques to gene expression data and gene‐related information in pediatric extracranial solid tumors. Our comprehensive search strategy encompassed terminology related to all forms of artificial intelligence as well as genomic and transcriptomic data. Importantly, our screening process, conducted using ASReview, was deliberately restricted to studies utilizing DL methodologies. This targeted approach yielded nine eligible studies that met our inclusion criteria (Table 2). According to our screening, neuroblastoma represented the predominant tumor type studied (6 out of 9 studies; Figure 3, Table 2). In contrast, only a single study each focused on pleuropulmonary blastoma, Wilms tumor/rhabdoid tumor, osteosarcoma, and a multi‐entity cohort including neuroblastoma, Non‐Hodgkin Lymphoma, rhabdomyosarcoma and Ewing sarcoma.

Neuroblastoma is a challenging entity due to its highly heterogeneous clinical behavior ranging from spontaneous regression to aggressive metastatic progression. High‐risk disease is still associated with poor outcomes [30]. This clinical relevance is reflected in the availability of large, publicly accessible gene expression datasets and gene‐related information, which likely explains the dominance of neuroblastoma in DL applications within this review. For neuroblastoma, there are several studies reporting gene‐based classifiers using classical machine learning models [4, 17, 31]. The performance metrics are promising in all of the mentioned studies. Yet, at least to our knowledge, only one of the published classifiers using classical ML is currently explored in a low‐risk neuroblastoma study protocol [17]. Specific advantages of DL could include potential reduction of required gene targets, inclusion of more and heterogenous datasets, utilization of unlabeled data and inclusion of differing forms of genetic information including gene expression but also mutation, methylation, and others [32].

While prior research has applied ML or DL to Wilms tumor imaging, few have examined gene‐related data. Only one study met our inclusion criteria for DL‐based gene expression analysis [29]. Given Wilms tumor's generally favorable prognosis (> 90% OS), there may be less clinical urgency to develop novel transcriptomic classifiers compared to entities like neuroblastoma [33]. For osteosarcoma, the DL literature is also richer in imaging applications [14, 34]. Whereas transcriptomic DL studies are rare, only one qualifying study was identified. Many other rare pediatric tumors remain uninvestigated through DL and gene‐related data studies, likely due to small sample sizes and fragmented data availability. By contrast, adult DL research spans nearly all major tumor types, including breast, lung, colorectal, liver, brain, and prostate cancers, and frequently utilizes large, pan‐cancer datasets [10, 35].

Most studies (6 out of 9) focused on supervised classification tasks, aiming to distinguish tumor subtypes or clinical stages based on gene expression data (Figure 3). Prognostic modeling, such as survival prediction, was reported in four studies, while biomarker discovery was featured in two. In contrast, adult DL research spans a broader set of applications, including therapy response prediction, mutational profiling, and multi‐omics integration [9, 10, 36, 37]. These additional applications may have potential to tailor the clinical process of pediatric oncology even more.

The spectrum of methodologies utilized in the included pediatric studies highlights the heterogeneity of computational strategies. Broadly, these approaches can be categorized into conventional DL models, classical ML methods, and ensemble architectures. In all nine included studies, a diverse range of DL techniques were applied, including standard DL and hybrid ML‐DL frameworks. This underscores the still exploratory nature of computational methods in pediatric surgical oncology.

Although most pediatric studies reported high internal performance (e.g., Cornero: 94%, Bandi: 93%, and Li: AUC 0.987), their generalizability is unclear due to the lack of standardized external validation. The clinical applicability of these models remains uncertain without independent testing and integration into workflows. Classification‐based DL tools may offer early or more precise diagnosis, but survival models are harder to validate in practice [16, 17]. Furthermore, technologies like intraoperative nanopore sequencing show promise for future surgical applications [38]. A key component of translating DL‐based techniques into clinical practice is the application of explainable DL, which enhances clinical understanding by adding transparency to “black box” applications. While techniques for explainability exist for genetic data, none of the included studies applied any such techniques.

Despite promising accuracy, key limitations persist in the included studies, including the use of small and heterogeneous cohorts (< 300 samples), poor documentation of preprocessing steps, and underreporting of hyperparameters or code availability. To compensate for small datasets, data augmentation approaches are being explored to artificially increase dataset sizes [39, 40, 41]. Furthermore, pediatric datasets often lack external benchmarking and suffer from sampling bias, a challenge also seen in adult oncology but increasingly addressed with harmonization efforts [18]. Notably, few studies conducted external validation using external datasets or prospective clinical cohorts. However, this is essential to progress to real‐world utility. Guidelines like TRIPOD and TRIPOD+AI help standardize evaluation and promote reproducibility, but none of the included articles adhered to these reporting guidelines [42]. Emerging techniques such as transfer learning and federated learning enable model training across distributed datasets without compromising data privacy, making them highly relevant in low‐sample pediatric contexts [43, 44, 45]. Future efforts should also include creation of shared repositories for gene expression with standardized data and outcomes.

Our review has some limitations. First, although we defined DL as neural networks with at least three hidden layers, varying definitions exist. Following this definition this would exclude more advanced networks as autoencoders or recurrent neural networks. However, upon re‐evaluation of excluded studies, no more advanced neural network architectures, such as autoencoders or recurrent neural networks, were excluded based on this criterion. Therefore, the impact of this methodological choice on the overall findings is likely limited. Next, the broad search strategy, aimed to include all relevant studies, led to a large number of studies to be included in the title/abstract screening. This potentially skewed the results, as ASReview led to 1000 consecutive irrelevant studies quite early on. Although the probability of missing relevant articles was low, especially since we also screened the references of the included articles, our inclusions still might not be exhaustive.

## 5 Conclusions

Ultimately, pediatric DL research has the potential to evolve beyond classification and survival prediction. DL applications for gene‐related data in pediatric solid tumors are promising yet underdeveloped compared to adult oncology. High internal accuracies in classification and early prognostic models demonstrate potential, but small datasets, limited tumor diversity, and lack of external validation hinder clinical translation. To bridge the pediatric‐adult gap, future directions should include pediatric AI consortia for gene‐related data, advanced modeling techniques (transfer learning, federated learning, and multi‐omics), rigorous, standardized validation and interdisciplinary collaboration.

## Author Contributions

**Simon Berhe:** conceptualization, methodology, formal analysis, investigation, writing – original draft, visualization. **Steffen E. Fuchs:** writing – review and editing, supervision. **Altuna Akalin:** writing – review and editing, supervision. **Alida F. W. van der Steeg:** writing – review and editing, supervision. **Steven W. Warmann:** writing – review and editing, supervision. **Myrthe A. D. Buser:** conceptualization, methodology, investigation, writing – original draft, visualization, supervision. **Moritz Markel:** conceptualization, methodology, formal analysis, investigation, writing – original draft, visualization.

## Funding

S.B. and S.E.F. are participants in the BIH‐Charité Clinician Scientist Program funded by the Charité—Universitätsmedizin Berlin and the Berlin Institute of Health. S.E.F. was supported during work by a fellowship from the Deutsche Forschungsgemeinschaft (DFG, German Research Foundation, Grant no. 439441203). This work was supported by the Deutsche Forschungsgemeinschaft (German Research Foundation) through the Collaborative Research Center “Decoding and Targeting Neuroblastoma Evolution” CRC1588 (Project no. 493872418) to S.B., S.E.F., and M.M.

## Conflicts of Interest

The authors declare no conflicts of interest.

## Supporting information


**Tables S1–S3:** Supporting Information.

**Table S4:** Supporting Information.

## Data Availability

The data supporting the findings of this study are available upon reasonable request from the corresponding author.
