# Supplementary material for: Deep Learning‐Based Analysis of Gene Expression Data and Gene‐Related Information in Pediatric Surgical Oncology: A Scoping Review
Source: Cancer Med. 2026 May 22;15(5):e71976. doi: 10.1002/cam4.71976 (PMC13240180; doi:10.1002/cam4.71976)
Supplement: Supplementary file 1 — Tables S1–S3: Supporting Information. [file CAM4-15-e71976-s002.docx]

**Supplementary Materials**

**Supplement table 1**

| PubMed | (("Artificial Intelligence"[Mesh] OR "Deep Learning"[Mesh] OR "Computational Biology"[Mesh] OR "Machine Learning"[Mesh] OR "Data Mining"[Mesh] OR "Biostatistics"[Mesh] OR Artificial Intelligen*[TIAB] OR CNN[TIAB] OR Machine Learn*[TIAB] OR Deep Learn*[TIAB] OR Deep-Learn*[TIAB] OR neural network*[TIAB] OR AI[TIAB] OR Bioinformatic*[TIAB] OR Biostatistic*[TIAB] OR Decision tree*[TIAB] OR Pattern recognition[TIAB] OR Support vector machine*[TIAB] OR Supervised learning[TIAB] OR Reinforcement learning[TIAB] OR Predictive model*[TIAB] OR Data mining[TIAB] OR Computational biology[TIAB] OR classif*[TIAB]))  AND  (("Child"[Mesh] OR "Pediatrics"[Mesh] OR Child*[TIAB] OR Pediatric*[TIAB] OR Paediatric*[TIAB] OR infant*[TIAB]))  AND  (("Neoplasms"[Mesh] OR Neoplasm*[TIAB] OR Cancer*[TIAB] OR oncolog*[TIAB] OR tumor*[TIAB] OR tumour*[TIAB] OR Malignan*[TIAB]))  AND  (("DNA"[Mesh] OR "RNA"[Mesh] OR RNA[TIAB] OR DNA[TIAB] OR "Methylation"[Mesh] OR "Genetics"[Mesh] OR "Chromosomes"[Mesh] OR "DNA Copy Number Variations"[Mesh] OR "Sequence Analysis"[Mesh] OR "Genomics"[Mesh]  OR Methylat*[TIAB] OR Genetic*[TIAB] OR Gene*[TIAB] OR Chromosom*[TIAB] OR "Copy number variation*"[TIAB] OR "Deoxyribonucleic acid"[TIAB] OR Sequenc*[TIAB] OR Genom*[TIAB] OR Muta*[TIAB] OR Phenotype*[TIAB] OR Genotype*[TIAB] OR "Polymerase chain reaction*"[TIAB] OR "Ribonucleic acid"[TIAB] OR epigene*[TIAB] OR proteomic*[TIAB] OR multi*omic*[TIAB] OR transcriptomic*[TIAB] OR proteogenomic*[TIAB] OR metabolomic*[TIAB] OR somatic*[TIAB] OR “clonal evolution”[TIAB])) |
| --- | --- |
| Scopus | (TITLE-ABS-KEY(Artificial Intelligen*) OR TITLE-ABS-KEY(CNN*) OR TITLE-ABS-KEY("Machine Learn*") OR TITLE-ABS-KEY("Deep Learn*") OR TITLE-ABS-KEY("neural network*") OR TITLE-ABS-KEY(AI)  OR TITLE-ABS-KEY(Bioinformatic*) OR TITLE-ABS-KEY(Biostatistic*) OR TITLE-ABS-KEY("Decision tree*") OR TITLE-ABS-KEY("Decision tree*") OR TITLE-ABS-KEY("Pattern recognition") OR TITLE-ABS-KEY("Support vector machine*") OR TITLE-ABS-KEY("Supervised learning") OR TITLE-ABS-KEY("Reinforcement learning") OR TITLE-ABS-KEY("Predictive modeling") OR TITLE-ABS-KEY("Data mining") OR TITLE-ABS-KEY(“Computational biology”) OR TITLE-ABS-KEY(classif*))  AND  (TITLE-ABS-KEY(Child*) OR TITLE-ABS-KEY(Pediatric*) OR TITLE-ABS-KEY(Paediatric*) OR TITLE-ABS-KEY(infant*))  AND  (TITLE-ABS-KEY("Neoplasms") OR TITLE-ABS-KEY(Neoplasm*) OR TITLE-ABS-KEY(Cancer*) OR TITLE-ABS-KEY(oncolog*) OR TITLE-ABS-KEY(tumor*) OR TITLE-ABS-KEY(tumour*) OR TITLE-ABS-KEY(Malignan*))  AND  (TITLE-ABS-KEY(RNA) OR TITLE-ABS-KEY(DNA) OR TITLE-ABS-KEY(Methylat*) OR TITLE-ABS-KEY(Genetic*) OR TITLE-ABS-KEY(Gene*) OR TITLE-ABS-KEY(Chromosom*) OR TITLE-ABS-KEY("Copy number variation*") OR TITLE-ABS-KEY("Deoxyribonucleic acid") OR TITLE-ABS-KEY(Sequenc*) OR TITLE-ABS-KEY(Genom*) OR TITLE-ABS-KEY(Muta*) OR TITLE-ABS-KEY(Phenotype*) OR TITLE-ABS-KEY(Genotype*) OR TITLE-ABS-KEY("Polymerase chain reaction*") OR TITLE-ABS-KEY("Ribonucleic acid") OR TITLE-ABS-KEY(epigene*) OR TITLE-ABS-KEY(proteomic*) OR TITLE-ABS-KEY(multi*omic*) OR TITLE-ABS-KEY(transcriptomic*) OR TITLE-ABS-KEY(proteogenomic*) OR TITLE-ABS-KEY(metabolomic*) OR TITLE-ABS-KEY(somatic*) OR TITLE-ABS-KEY(Clonal evolution)) |
| Embase | (('artificial intelligence'/exp OR 'convolutional neural network'/exp OR 'machine learning'/exp OR 'deep learning'/exp OR 'bioinformatics'/exp OR 'biostatistics'/exp OR 'data mining'/exp  OR ‘Artificial Intelligen*’:ti,ab,kw OR CNN:ti,ab,kw OR 'Machine Learn*':ti,ab,kw OR 'Deep Learn*':ti,ab,kw OR 'Deep-Learn*':ti,ab,kw OR 'neural network*':ti,ab,kw OR AI:ti,ab,kw  OR 'Bioinformatic*':ti,ab,kw OR 'Biostatistic*':ti,ab,kw OR 'Decision tree*':ti,ab,kw OR 'Decision tree*':ti,ab,kw OR 'Pattern recognition':ti,ab,kw OR 'Support vector machine*':ti,ab,kw OR 'Supervised learning':ti,ab,kw OR 'Reinforcement learning':ti,ab,kw OR 'Predictive modeling':ti,ab,kw OR 'Data mining':ti,ab,kw OR 'Computational biology':ti,ab,kw OR ‘Classif*’:ti,ab,kw)  AND  ('pediatric'/exp OR 'childhood'/exp  OR ‘Child*’:ti,ab,kw OR ‘Pediatric*’:ti,ab,kw OR ‘Paediatric*’:ti,ab,kw OR ‘infant*’:ti,ab,kw)  AND  ('malignant neoplasm'/exp OR 'oncology'/exp  OR 'Neoplasms':ti,ab,kw OR Neoplasm*:ti,ab,kw OR Cancer*:ti,ab,kw OR oncolog*:ti,ab,kw OR tumor*:ti,ab,kw OR tumour*:ti,ab,kw OR Malignan*:ti,ab,kw)  AND  ('RNA'/exp OR 'DNA'/exp OR 'methylation'/exp OR 'genetic*'/exp OR 'gene*'/exp OR 'gene sequenc*'/exp OR 'gene mutation*'/exp OR 'phenotype*'/exp OR 'genotype*'/exp OR 'pcr'/exp  OR ‘RNA’:ti,ab,kw OR ‘DNA’:ti,ab,kw OR Methylat*:ti,ab,kw OR ‘Genetic*’:ti,ab,kw OR ‘Gene*’:ti,ab,kw OR ‘Chromosom*’:ti,ab,kw OR 'Copy number variation*':ti,ab,kw OR 'Deoxyribonucleic acid':ti,ab,kw OR ‘Sequenc*’:ti,ab,kw OR ‘Genom*’:ti,ab,kw OR ‘Muta*’:ti,ab,kw OR ‘Phenotype*’:ti,ab,kw OR ‘Genotype*’:ti,ab,kw OR 'Polymerase chain reaction*':ti,ab,kw OR 'Ribonucleic acid':ti,ab,kw OR ‘epigene*’:ti,ab,kw OR ‘proteomic*’:ti,ab,kw OR ‘multi*omic*’:ti,ab,kw OR ‘transcriptomic*’:ti,ab,kw OR ‘proteogenomic*’:ti,ab,kw OR ‘metabolomic*’:ti,ab,kw OR ‘somatic*’:ti,ab,kw OR ‘clonal evolution’:ti,ab,kw)) |

**Supplement table 2**

| **Database** | **Selected article types** | **Hits** |
| --- | --- | --- |
| Pubmed | Clinical trial, meta-analysis, randomized controlled trial, review, systematic review | 1,860 |
| Scopus | Article, review | 14,262 |
| Embase | Article, review, article in press | 7,535 |
| TOTAL | - | 23,657 |

**Supplement table 3**

| TOTAL | 23,657 |
| --- | --- |
| Duplicates removed | 7,299 |
| Final dataset | 16,358 |
