# Supplementary material for: Deep Learning‐Based Analysis of Gene Expression Data and Gene‐Related Information in Pediatric Surgical Oncology: A Scoping Review
Source: Cancer Med. 2026 May 22;15(5):e71976. doi: 10.1002/cam4.71976 (PMC13240180; doi:10.1002/cam4.71976)
Supplement: Supplementary file 2 — Table S4: Supporting Information. [file CAM4-15-e71976-s001.docx]

**Supplementary Table -** PROBAST risk-of-bias assessment of included studies.

| **Study** | **n** | **D1 Participants** | **D2 Predictors** | **D3 Outcome** | **D4 Analysis** | **Overall ROB** |
| --- | --- | --- | --- | --- | --- | --- |
| **Bandi & Santhisri** 2023^21^ | NR | **H** — Sample size unreported; no inclusion criteria; PPB is extremely rare | **H** — Preprocessing undocumented; no feature selection rationale | **H** — Ground truth source not described | **H** — Single 80/15/5 split; no CV; no external validation; accuracy only (96%) | **H** |
| **Cornero et al.** 2012^22^ | 182 | **U** — Platform-restricted convenience sample; 4 institutions | **H** — Probeset assignment on full dataset before split; DS2 dual-use leakage | **L** — 5-year OS clearly defined; adequate follow-up | **H** — 726 classifiers tested without correction; no AUC; hyperparameters unreported | **H** |
| **Feng et al.** 2021^23^ | 721 | **L** — 498 training + 223 independent external cohort; well-documented | **H** — 172-gene chi-square selection performed on full dataset before splitting | **L** — OS clearly defined; time-dependent ROC at 3, 5, 10 years | **H** — Training AUC 0.968 vs. test 0.891; specificity 0.944 vs. 0.605; epochs/batch unreported | **H** |
| **Li et al.** 2023^24^ | ~71 | **H** — ~71 training samples; multi-platform batch-corrected; authors acknowledge small size | **H** — DEG + RF feature selection on full dataset; binarization discards continuous data | **L** — Diagnostic outcome from GEO tissue labels | **H** — Training AUC = 1.0; no CV for ANN; ANN architecture unreported | **H** |
| **Pal et al.** 2007^25^ | 88 | **H** — 63 training samples; NHL class n=8; cell lines mixed with biopsies | **H** — Multi-stage data-driven feature selection; learning rates unreported | **L** — Established histopathological four-class SRBCT classification | **H** — No CV; single fixed split; 100% accuracy; no AUC or per-class metrics | **H** |
| **Park & Nam** 2019^26^ | 280 | **U** — Single GEO dataset; class distribution per INSS stage not reported | **H** — No feature selection; 13,091 genes fed to DNN with ~224 training samples | **U** — Post-surgical INSS stage; clinical utility from pre-treatment expression unclear | **H** — Training accuracy 100% vs. test 55.6%; no CV; no external validation | **H** |
| **Su et al.** 2023^27^ | N/A | **H** — No patient cohort; lncRNA–disease database pairs (not gene expression data) | **H** — Graph-derived features; DNN/LightGBM hyperparameters unreported | **H** — Computational association — not a clinical outcome | **H** — No external validation; verification bias (unknown ≠ negative); case study only | **H** |
| **Tranchevent et al.** 2019^28^ | 678 | **U** — Four public cohorts; Maris cohort follow-up only 2.3 years | **H** — Patient Similarity Networks constructed including test samples; potential leakage | **U** — Binary survival ignores censoring; no time-to-event modelling | **U** — Hyperparameters fully reported; multi-dataset external validation; Maris bACC 53% (near-random) | **H** |
| **Hosseiniyan Khatibi et al.** 2023^29^ | NR | **H** — Sample size unreported; rhabdoid tumor is extremely rare | **H** — Multi-step data-driven feature selection; methods not fully accessible | **L** — Clear diagnostic categories (Wilms tumor vs. rhabdoid tumor) | **H** — mRNA test AUC (97%) exceeds training AUC (94%) — unusual; no external validation | **H** |

**Abbreviations:** D1–D4, PROBAST domains 1–4; H, high risk of bias; L, low risk of bias; U, unclear risk of bias; N/A, not applicable; NR, not reported; n, number of participants; OS, overall survival; AUC, area under the receiver operating characteristic curve; CV, cross-validation; DEG, differentially expressed genes; RF, random forest; DNN, deep neural network; ANN, artificial neural network; PPB, pleuropulmonary blastoma; SRBCT, small round blue cell tumors; INSS, International Neuroblastoma Staging System; bACC, balanced accuracy; ROB, risk of bias.

**Note:** Su et al.27 uses lncRNA–disease association databases rather than patient-level gene expression data; standard PROBAST criteria are of limited applicability for this study. All studies used retrospectively collected data. Colour coding: **H** = high, **U** = unclear, **L** = low risk of bias.
